# Malakoplakia of the Kidney Transplant

**DOI:** 10.5334/jbsr.4173

**Published:** 2026-01-13

**Authors:** Emiel Declerck, Annelies Laerte, Henri Vandermeulen

**Affiliations:** 1 Department of Radiology, UZ Leuven, Leuven, Belgium

**Keywords:** malakoplakia, kidney transplant, ultrasound, MRI, urogenital radiology

## Abstract

*Teaching point:* Malakoplakia is a rare but clinically significant condition in immunocompromised patients and in these patients to be included in the differential diagnosis to distinguish this pseudotumoral process in a renal graft from malignant entities such as renal cell carcinoma and post-transplant lymphoproliferative disease (PTLD).

## Case

A 66-year-old female was referred to the emergency department. She reported several days of fever, hypogastric pain, and skin erythema around the renal transplant surgical scar. A renal transplantation had been performed seven years earlier. Clinical examination confirmed the local erythema and mild tenderness on palpation.

**Figure 1:**
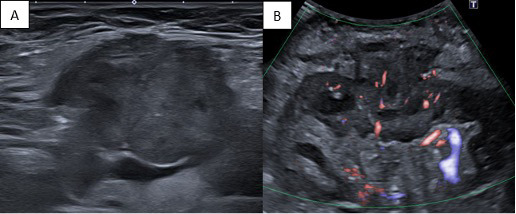
Ultrasound of malakoplakia in the transplanted kidney showing a hypoechoic **(A)**, hypervascular **(B)** mass in the interpolar region.

Laboratory tests demonstrated impaired renal function and elevated inflammatory markers. Ultrasound of the kidney demonstrated a hypoechoic, hypervascular mass in the interpolar region, approximately 3 cm in diameter (Figure 1).

**Figure 2:**
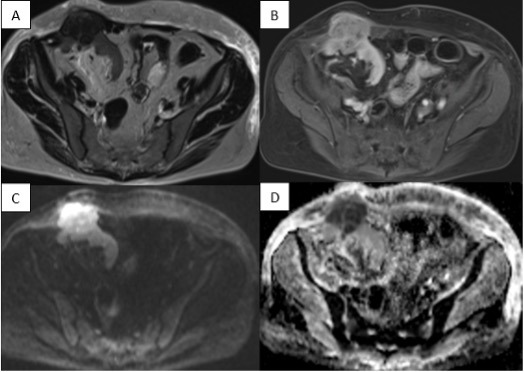
MRI of the transplanted kidney showed a T2-hypointense lesion **(A)** with enhancement after gadolinium contrast administration on the T1 fat-saturated sequence **(B)** and restricted diffusion **(C, D)**.

Further assessment by MRI confirmed the presence of the mass and also identified two smaller lesions with identical imaging characteristics. The lesions appeared T2-hypointense and T1FS-hyperintense, and demonstrated restricted diffusion and contrast enhancement (Figure 2). Furthermore, the lesions were largely progressive compared with the ultrasound performed two weeks earlier, with infiltration of the abdominal wall.

**Figure 3:**
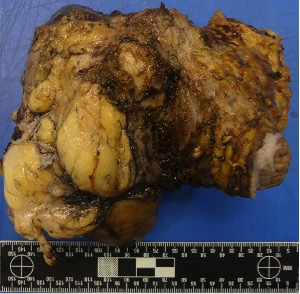
Macroscopic appearance of malakoplakia in the transplanted kidney.

Based on these features, the differential diagnosis included renal cell carcinoma, post-transplant lymphoproliferative disorder (PTLD), and chronic inflammatory disease. An ultrasound-guided percutaneous biopsy of the dominant lesion confirmed the diagnosis of malakoplakia of the renal graft. Despite targeted intravenous antibiotic therapy, control of the infection was not achieved, and a transplantectomy was performed. Macroscopic examination showed multifocal, multinodular, yellowish renal masses with purulent and necrotic appearance (Figure 3).

## Comment

Malakoplakia is a rare granulomatous disease resulting from an abnormal response to infection, predominantly in immunocompromised patients. Although the bladder is most commonly affected, other organs (including renal grafts) may also be involved. *Escherichia coli* and other Gram-negative bacilli are the primary pathogens in genitourinary malakoplakia. Histologically, the disease is characterized by histiocytic infiltrate containing cytoplasmic inclusions (Michaelis–Gutmann bodies).

On imaging, ultrasound typically shows a hypoechoic, poorly defined lesion. MRI demonstrates T2-hypointensity, T1-hyperintensity, heterogeneous contrast enhancement, and restricted diffusion. PET-CT reveals pronounced FDG-avidity. A definitive diagnosis generally requires a targeted biopsy.

The differential diagnosis includes PTLD and renal cell carcinoma. Rapid onset and progression favor malakoplakia. Accurate distinction between these entities is often not possible without histological confirmation.

First-line management consists of broad-spectrum intravenous antibiotics and supportive therapy. In refractory cases, surgical excision of the affected organ may be required to achieve disease control [1].
